# Feasibility evaluation of intravoxel incoherent motion diffusion-weighted imaging in the diagnosis of skull-base invasion in nasopharyngeal carcinoma

**DOI:** 10.7150/jca.80679

**Published:** 2023-01-09

**Authors:** Weiquan Wu, Jun Xia, Bin Li, Wenci Liu, Zhan Ge, Zhi Tan, Qiujin Bu, Wubiao Chen, Yuange Li

**Affiliations:** 1 Clinical Research Experiment Center, Affiliated Hospital of Guangdong Medical University, Zhanjiang, Guangdong, China.; 2 Department of Radiology, Affiliated Hospital of Guangdong Medical University, Zhanjiang, Guangdong, China.

**Keywords:** Nasopharyngeal carcinoma (NPC), skull-base invasion (SBI), intravoxel incoherent motion diffusion-weighted imaging (IVIM-DWI), magnetic resonance imaging (MRI)

## Abstract

**Objective:** This study aimed to evaluate the feasibility of intravoxel incoherent motion diffusion-weighted imaging (IVIM-DWI) in the diagnosis of skull-base invasion (SBI) in nasopharyngeal carcinoma (NPC).

**Materials and methods:** A total of 50 patients pathologically diagnosed with NPC and a group of 40 controls comprised of those with either normal nasopharynx or patients with nasopharyngitis underwent conventional MRI and IVIM-DWI scans with 3 different groups of b values. Among the 50 patients, 36 patients diagnosed with SBI in NPC were included in the case group according to SBI criteria. All subjects (including those in the control group and case group) were divided into the b1, b2, and b3 groups based on their b values. The pure diffusion coefficient (D), perfusion-related incoherent microcirculation (D*), and microvascular volume fraction (f) values obtained in each measurement area of each group were tested for variance. Next,2 groups of b-value parameters with statistically significant data in the 3 groups were randomly selected for use in both the control group and the case group. A *t-*test was performed on the D, D*, and f values obtained by measuring each area of the skull base, and the area under the curve (AUC) of the receiver operating characteristic (ROC) was used to evaluate the diagnostic efficacy of the D, D*, and f values.

**Results:** There was no statistical significance among the D, D*, and f values of the b1 and b3 groups (*P*>0.05), and the differences in parameters between the b1 and b2 groups were statistically significant(*P* < 0.05),and the differences in parameters between the b3 and b2 groups were also statistically significant(*P* < 0.05).The f value of the case group, which was obtained using the b1 and b2 parameters in each area of the skull base, was lower than that of the control group (*P* <0.05).The D, D*, and f values of the case group obtained by the b1 and b2 parameters in the pars petrosa of the temporal bone (including the foramen lacerum) were lower than those of the control group (*P*<0.05).When the parameters of the b1 group were used in the corpus of sphenoid bone (including the foramen ovale), the D, D*, and f values of the control group and the case group were compared, yielding a statistically significant difference (*P*<0.05).When the parameters of the b1 group were used, the diagnostic efficacy of the f value in each area of the skull base was the highest (AUC=0.908-0.991), followed by the D* value (AUC=0.624-0.692).

**Conclusion:** When the number of b values <200 s/mm^2^ in IVIM-DWI accounts for more than half of the selected b values, IVIM-DWI is highly stable for the diagnosis of SBI in NPC. The D, D*, and f values of the bone and muscle areas of the skull base in patients with SBI of NPC showed a downward trend, and the f value had the best diagnostic performance, followed by the D* value, while the D value had the worst. Thus, IVIM-DWI can be used as a noninvasive method in the diagnosis of SBI in NPC.

## Introduction

Nasopharyngeal carcinoma (NPC) is a common malignant tumor with a certain regional distribution, mainly in southern China and Southeast Asia 1. In recent years, the early detection and timely treatment of NPC have significantly improved the 5-year survival rate and quality of life of patients with NPC. To a certain extent, these achievements rely on imaging methods for the accurate staging of NPC before treatment, which in turn provides a basis for clinicians to formulate accurate treatment plans 2. The occurrence of NPC is relatively insidious, and most patients already have cervical lymph node and skull-base invasion (SBI) when they are examined. The incidence rate of SBI of NPC has been reported to be 65.51%, which is relatively high 3. SBI of NPC directly affects the diagnosis and correct staging of NPC, which in turn affects the formulation of the diagnosis of and treatment strategies for NPC, and thus the prognosis of patients 4, 5.

The gold standard for the diagnosis of SBI in NPC is still pathological biopsy. However, due to the particularity of the anatomical site of the skull base, it is difficult to obtain samples by pathological biopsy, which limits the application of pathological biopsy. Thus, various imaging techniques are used for SBI of NPC, such as computed tomography (CT), positron emission tomography (PET-CT), and magnetic resonance imaging (MRI). However, CT and PET-CT are radioactive, and do not clearly display the anatomical structure of the skull base, while MRI has the advantages of no radiation and high resolution of skull base anatomy, which makes radiation-free MRI the more favorable option 6. With the continuous and rapid development of MRI technology, various MRI functional sequences have gradually matured, most notably conventional MRI, dynamic contrast-enhanced MRI (DCE-MRI), traditional diffusion-weighted imaging (DWI), and intravoxel incoherent motion DWI (IVIM-DWI) 7-9. Some studies have applied DCE-MRI to the diagnosis of NPC, but DEC-MRI requires a contrast agent, which has limitations for patients with a history of allergies 10. Some studies have applied DWI to the diagnosis of SBI in NPC, but DWI is affected by susceptibility artifacts and low resolution, which easily leads to image distortion and deformation, especially in the skull-base region 11.

IVIM-DWI is a new imaging method that can observe the microscopic motion of voxels without a contrast agent. It reflects the diffusion of water molecules and microcirculation perfusion simultaneously based on a bi-exponential mathematical model and is a feasible method for diagnosing NPC 12. Thus far, several studies have applied IVIM-DWI to the staging and prognostic evaluation of NPC, but very few have applied this sequence to the diagnosis of SBI of NPC, and no consensus has been reached as to the b value of IVIM-DWI 13, 14. Therefore, we sought to evaluate the feasibility of selecting the appropriate b value of IVIM-DWI for the diagnosis of SBI in NPC.

## Materials and Methods

### Patients

The control group comprised 40 volunteers with normal nasopharynx or patients with nasopharyngitis treated at the Affiliated Hospital of Guangdong Medical University from January 2021 to May 2022. The control group patients were aged 35-90 years, with 22 being male and 18 female.

The total of 50 NPC patients treated at the Department of Oncology, Affiliated Hospital of Guangdong Medical University from January 2021 to May 2022.All of the patients were pathologically diagnosed with NPC according to the diagnostic criteria of the Chinese Medical Association. Among the 50 patients, 36 patients diagnosed with SBI in NPC were included in the case group according to SBI criteria. The case group patients were aged 32-78 years, with 26 being male and 10 female.

The main clinical symptoms and signs of SBI in NPC were epistaxis (26 cases), hearing impairment (24 cases), nasal obstruction (18 cases), headache (16 cases), facial numbness (5 cases), diplopia and eye symptoms (5 cases), and cervical lymph nodes (21 cases). None of the patients had received radiotherapy, chemotherapy, or surgical treatment before examination (Table 1).

The study was approved by the Ethics Committee of The Affiliated Hospital of Guangdong Medical University. All the methods described here were performed in accordance with the relevant guidelines and regulations. Informed consent forms were signed by all patients.

### Exclusion and inclusion criteria for SBI in NPC

Based on the relevant literature 15, 16, the inclusion criteria for SBI of NPC were as follows: (1) a pathological diagnosis of NPC; and (2) an imaging diagnosis of SBI based on a low signal intensity defect of the bone cortex on MRI, high signal intensity bone marrow replaced by low signal intensity tissue on MRI, skull-base muscle signal changes, and an unclear demarcation between skull-base muscle and the primary tumor. The images had to show the invasion of 1 or more of the following sites: the pterygopalatine fossa, infratemporal fossa, sinus sphenoidalis, sellar floor, clivus, pars petrosa of the temporal bone, foramen ovale, foramen rupture, cavernous sinus, or jugular regions. Patients were excluded from the study if they met any of the following exclusion criteria: (1) had claustrophobia, a pacemaker, or severe liver and kidney insufficiency, or were in a coma, or had other symptoms; (2) had images of a quality that could not be assessed; (3) had other primary malignant tumors; and/or (4) did not have SBI according to the above SBI criteria.

### Imaging methods

The MRI equipment used included a GE 3.0T (GE Healthcare, Piscataway, NJ, USA) or GE 1.5T (GE Healthcare, Piscataway, NJ, USA) magnetic resonance apparatus and an 8- or 16-channel-phased array head and neck combined coil. Each patient was placed in a supine position with both hands on either side of the body and instructed not to swallow. The scanning range was from the upper border of the auricle to the level of the lower border of the cervical 3 vertebrae in the transverse axis, and the sagittal and coronal planes included the bilateral supraclavicular fossa. Conventional MRI sequences included transverse axial short-tau inversion recovery (STIR) and T1-weighted imaging(T1WI), coronal T2-weighted imaging (T2WI), sagittal T1WI, and T1WI-enhanced scan sequences (including transverse axial, coronal, and sagittal images). The IVIM-DWI sequence made 3 sets of data for each patient according to the different b values. The b1 values were 0, 10, 30, 50, 100, 150, 200, 600, and 1000(s/mm^2^). The b2 values were0, 50, 100, 150, 200, 600, 800, 1000, and 1200 (s/mm^2^). The b3 values were0, 10, 20, 30, 50, 100, 200, 600, and 1000 (s/mm^2^). The contrast agent was Gadolinium diethylene triamine pentaacetic acid (Gd‑DTPA) at a dose of 0.1 mmol/kg.

### Image analysis

The IVIM-DWI data were transferred to the GE Healthcare postprocessing workstation, and Function-MADC software was used to postprocess the images. IVIM-DWI images with different multiple b values for each participant were selected for the double exponential model calculation to obtain pseudo-color images of IVIM-DWI quantitative parameters (i.e., the pure diffusion coefficient[D], perfusion-related incoherent microcirculation[D*],and microvascular volume fraction[f] values).Next, combined with conventional MRI sequences, the regions of interest (ROIs) of the skull-base muscles, skull-base bones, and primary lesions of the control group and the SBI of the NPC group were each delineated on the pseudo-color images to obtain the D, D*, and f values. The ROI range was about 0.5-1.0 cm^2^. Each ROI of the lesion and the anatomical structure were sketched 3 times, with care being taken to avoid sketching the surrounding large blood vessels.

SBI includes the pterygopalatine fossa, infratemporal fossa, sinus sphenoidalis, sellar floor, clivus, pars petrosa of the temporal bone, foramen ovale, foramen lacerum, sinus cavernous, and jugular vein area 17. Among them, the sinus cavernous and jugular vein area were excluded from this study, as these are vascular areas, and there may be errors in their measurement. For the convenience of measurement, the remaining structures were artificially divided into the following 5 measurement areas: the pterygopalatine fossa/processus pterygoidei, the infratemporal fossa, the sinus sphenoidalis/sellar floor/clivus, the pars petrosa of the temporal bone (including foramen lacerum), and the corpus of sphenoid bone (including foramen ovale).

### Statistical analysis

The selected participants (including those in the control group and the case group) were allocated into the b1, b2, and b3 groups based on the different b values. The D, D*, and f values obtained in each measurement area of each group were tested for variance. Next, 2 groups of b-value parameters with statistically significant data in the 3 groups were randomly selected for use in both the control group and the case group. A*t*test was conducted on the D, D*, and f values obtained by measuring each area of the skull base, and the areas under the curve (AUC) of the receiver operating characteristic (ROC) was used to evaluate the diagnostic efficacy of the D, D*, and f values. Statistical analysis was performed using SPSS17.0 software (SPSS, Inc., Chicago, IL, USA). A *P* value < 0.05 indicated a statistically significant difference.

## Results

### General information of enrolled cases

In the control group, 6 cases of nasopharyngitis (all pathologically confirmed) and 3 cases of paranasal sinus inflammation were found, with the remaining cases being considered normal. In the case group, the invasion occurred in the pterygopalatine fossa/processus pterygoidei in 23 cases (63.9%, 23/36), infratemporal fossa in 1 case (2.7%, 1/36), sinus sphenoidalis/sellar floor/clivus in 26 cases (72.2%, 26/36), the pars petrosa of the temporal bone (including foramen lacerum) in 24 cases (66.7%, 24/36), and corpus of sphenoid bone (including the foramen ovale) in 15 cases (41.7%, 15/36). As there were too few cases of infratemporal fossa involvement, this part was excluded from the subsequent statistical analyses.

### Images of MRI routine sequence

**Figure 1:**
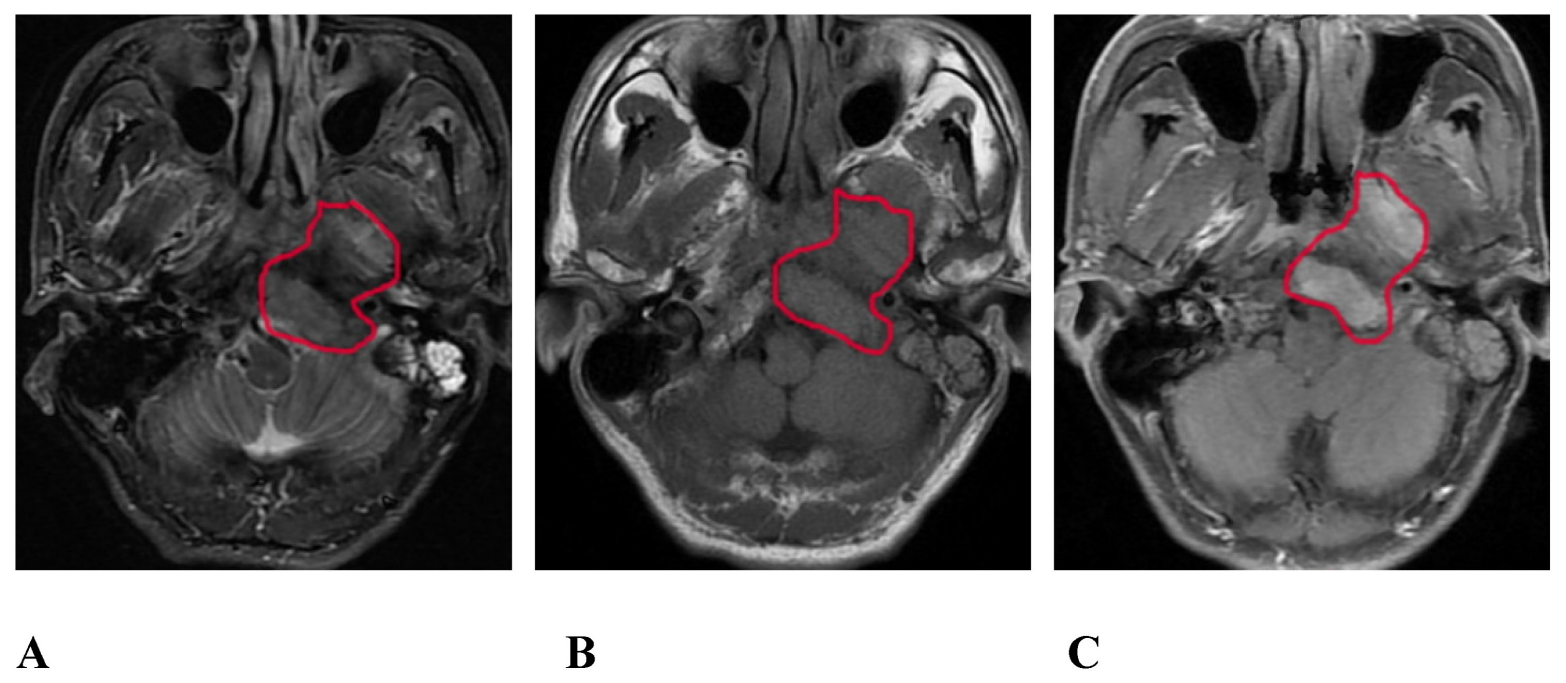
A 57-year-old male with SBI in NPC. A, B, and C show the conventional MRI sequence transverse axial STIR, T1WI, and T1WI-enhanced scan sequences, respectively. The red circle shows the clivus, the left processus pterygoidei, and the parapharyngeal muscle involvement. STIR is slightly hyperintense, T1WI is isointense, and the enhanced scan is significantly enhanced. SBI, skull-base invasion; NPC, nasopharyngeal carcinoma; STIR, short-tau inversion recovery; T1WI, T1-weighted imaging.

On the routine MRI sequence, bone SBI manifests as bone marrow signal changes or the osteolytic destruction of bone with a low signal on T1WI, a high signal on STIR, and uneven and obvious enhancement on the enhanced scan (Figure 1); meanwhile, muscle SBI manifests as changes in the shape and signal of the muscle such that the signal of the involved muscle is increased on the STIR sequence, and the corresponding area on the enhanced scan shows patchy and uneven enhancement.

### Images of IVIM-DWI sequence

**Figure 2:**
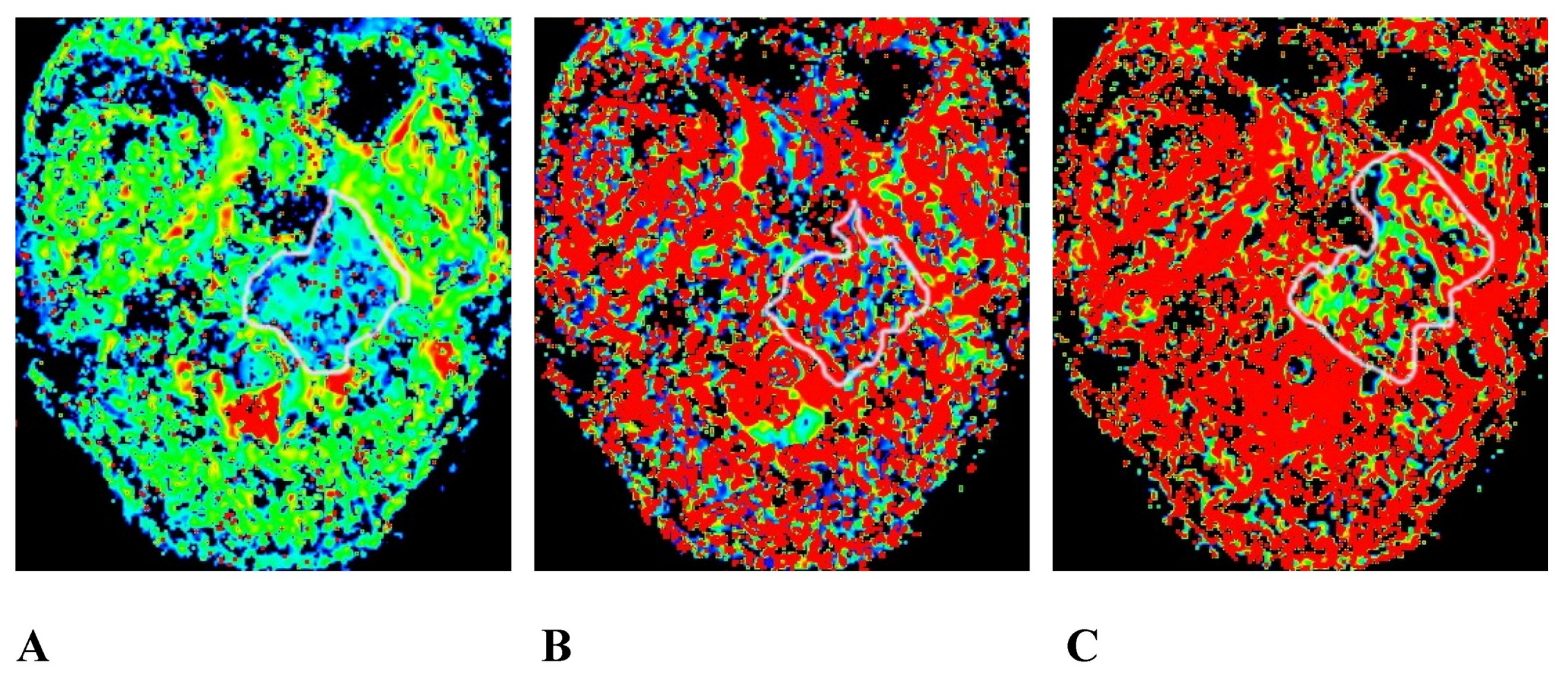
The same patient as that shown in Figure 1. A, B, and C are the pseudo-color images of D, D*, and f, respectively, obtained by postprocessing the IVIM-DWI using the b value of group b1. The white circle shows the involvement of the clivus, the left processus pterygoidei, and the parapharyngeal muscles. The D, D*, and f values are all decreased, and the f pseudo-color map shows the largest involvement range. IVIM-DWI, intravoxel incoherent motion diffusion-weighted imaging.

**Figure 3:**
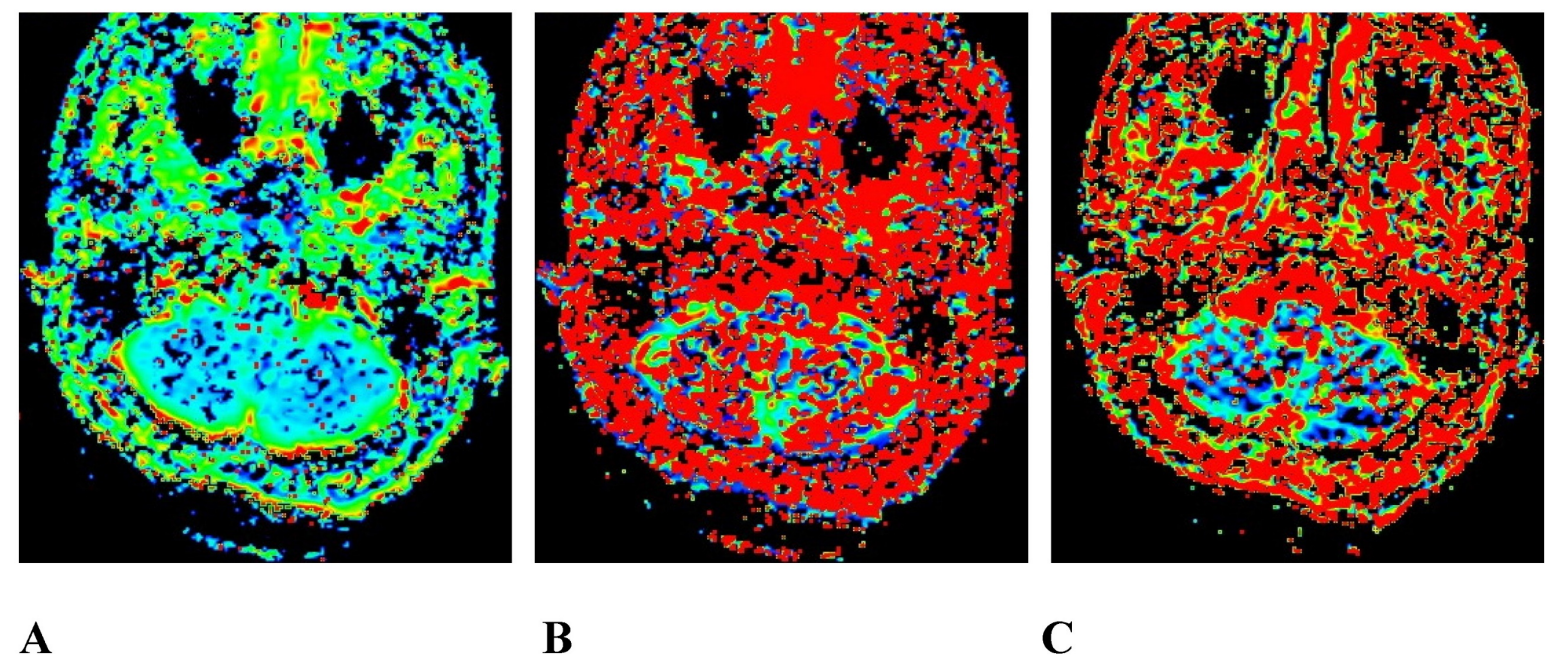
A 45-year-old male pathologically diagnosed with nasopharyngeal inflammation. A, B, and C are the pseudo-color images of D, D*, and f, respectively, obtained by postprocessing the IVIM-DWI using the b value of group b1, all of which show a uniform and symmetrical color distribution of the paranasal pharynx and skull base. IVIM-DWI, intravoxel incoherent motion diffusion-weighted imaging.

We obtained 3 sets of original images with different b values for each participant and sent them to the GE Healthcare postprocessing workstation. The images were postprocessed with Function-MADC software to obtain the D, D*, and f pseudo-color images (Figures 2 and 3).

### Analysis of IVIM parameters

The comparison of the D, D*, and f values in the b1, b2, and b3 groups indicated that there were no statistically significant differences in the parameters between the b1 and b3 groups (*P*>0.05), while the parameters of the b1 and b3 groups were statistically significant compared to those of the b2 group (*P*<0.05) (Table 2). The comparison of the D, D*, and f values of each area of the skull base in the control group and the case group obtained by the b value parameters of the b1 group showed that the D, D*, and f values of the control group in the pars petrosa of the temporal bone (including foramen lacerum) and the corpus of sphenoid bone (including the foramen lacerum) were significantly higher than the case group (*P* < 0.05), and the f values of the control group in each area of the skull base were significantly higher than the case group (*P* < 0.05) (Table 3).

The comparison of the D, D*, and f values of each area of the skull base in the control group and the case group obtained by the b value parameters of the b2 group showed that the D, D*, and f values of the control group in the pars petrosa of the temporal bone (including foramen lacerum) were significantly higher than the case group (*P* < 0.05), and the f values of the control group in each area of the skull base were significantly higher than the case group (*P* < 0.05) (Table 4).

**Figure 4:**
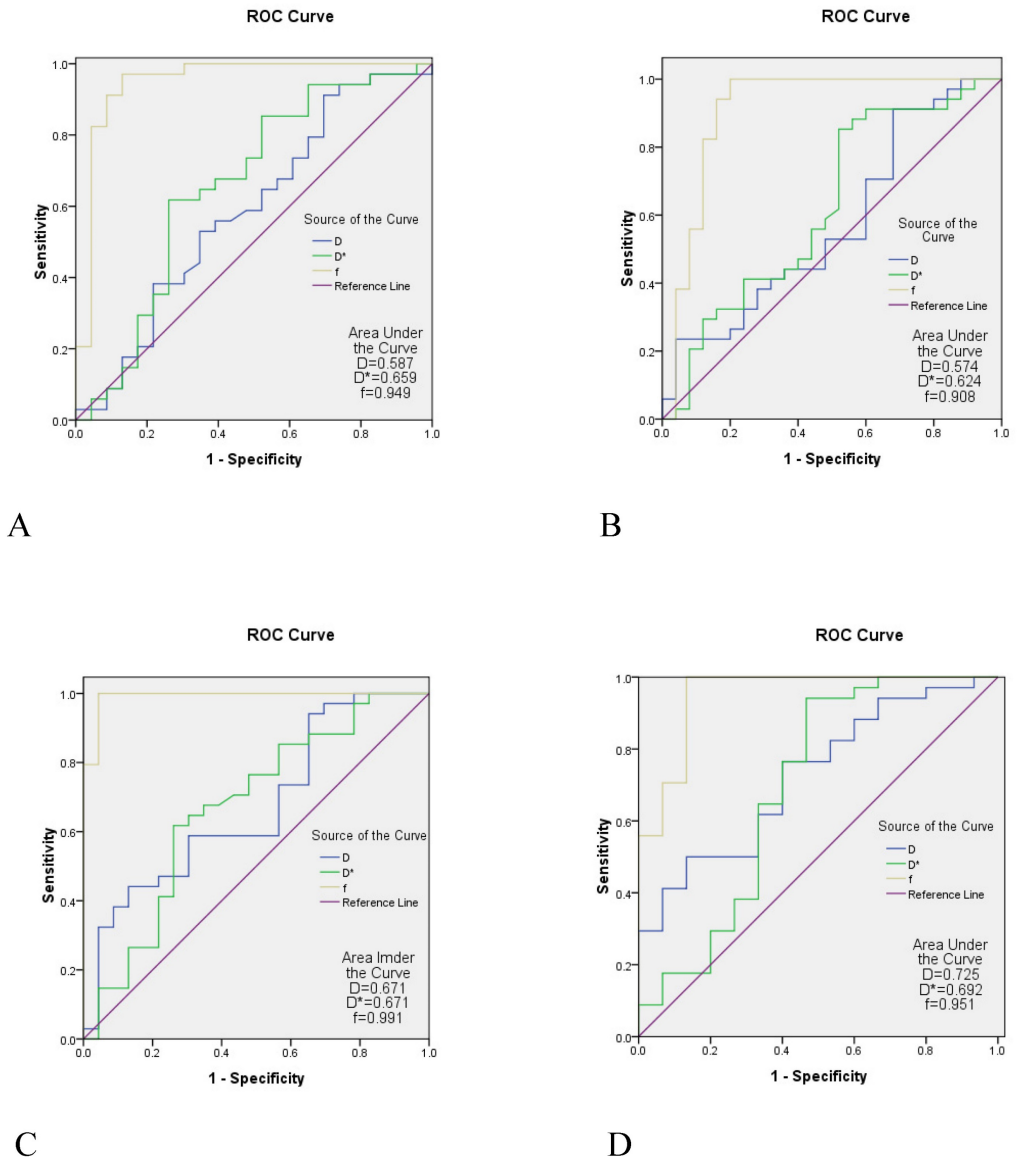
The ROC curve analysis of IVIM. A is the ROC curve of the the pterygopalatine fossa/processus pterygoidei, B is the ROC curve of the sinus sphenoidalis/sellarfloor/clivus; C is the ROC curve of the pars petrosa of the temporal bone (including the foramen lacerum), D is the ROC curve of the corpus of sphenoid bone (including foramen the ovale). The diagnostic efficacy of the f value in each region of the skull base appeared to be the highest (AUC = 0.908-0.991), the diagnostic efficacy of the D* value was the second highest (AUC = 0.624-0.692), and the diagnostic efficacy of the D value was slightly lower (AUC = 0.574-0.725). ROC, receiver operating characteristic; IVIM-DWI, intravoxel incoherent motion diffusion-weighted imaging; AUC, area under the curve.

A comparison between the control group and the case group was strong when the parameters of the b1 group were used. The AUC of the f value in each area of the skull base was 0.908-0.991, the AUC value of the D* value was 0.624-0.692, and the AUC of the D value was slightly lower (0.574-0.725) (Table 5 and Figure 4).

## Discussion

IVIM-DWI, as a bi-exponential model with multiple b values, separates the diffusion motion of pure water molecules in tissues from capillary microcirculation perfusion. It not only provides quantitative parameters of the water molecule motion-pure diffusion coefficient (D), which represents the diffusion motion of pure water molecules, but also reflects the tissue perfusion-pseudo-diffusion coefficient (D*) and diffusion score(f). D*represents the incoherent movement of the microcirculation in the tissue, which is affected by the capillary morphology and blood flow velocity in the tissue, while f represents the volume ratio of the diffusion effect related to microcirculation perfusion to the total diffusion effect, which is related to the blood volume of capillaries, with its size ranging from 0 to 1 18, 19.

At present, IVIM-DWI is used in the differential diagnosis of tumors in multiple organs of the body, such as the head and neck 20, breast 21, lung 22, liver 23, pancreas 24, and kidney 25. Studies have shown that IVIM-DWI has diagnostic value in the differential diagnosis and staging of NPC and can be used as a potential noninvasive imaging diagnostic method for the clinical evaluation of NPC 26, 27. These findings formed the basis of this study in applying this sequence to the diagnosis of SBI in NPC.

The patients were divided into 3 groups based on the different b values in IVIM-DWI, and statistical analysis showed that there was no statistical significance between the parameters of b1 and b3 groups (*P*>0.05), while the parameters of the b1 and b3 groups were statistically significant compared to those of the b2 group (*P*<0.05). The reason for the above statistical analysis is that the number of the b values >200 s/mm^2^ in the b1 and b3 groups were the same; the number of the b values<200 s/mm^2^ reached 6, but in the group b2, the number of the b values>200 s/mm^2^ was 5, while the number of the b values<200 s/mm^2^ was 4.So when the number of the b values<200 s/mm^2^ reaches a certain number in the selected b value, it shows that the repeatability of the IVIM-DWI of SBI in NPC is extremely high.

As the parameters of b1 and b3 groups were not statistically different, the D, D*, and f values of each area of the skull base in the control group and the case group obtained by the b value parameters of the b1 and b2 groups were randomly compared. The f value of the case group and the D, D*, and f values of the pars petrosa of the temporal bone (including the foramen lacerum) obtained by using the b1 and b2 parameters in each area of the skull base were lower than those of the control group (*P* < 0.05). This is consistent with the results obtained by Lai 28, who applied IVIM-DWI to different stages of NPC. Lai compared the D, D*, and f values of different stages and found that the D, D*, and f values of the T3 and T4 stage cases were lower than those of the T1 and T2 stage cases (*P*<0.05). Similarly, Zhang 29 applied IVIM-DWI to the differential diagnosis of NPC and adenoid hypertrophy, reporting that the D and f values of the NPC group were lower than those of the adenoid hypertrophy group.

In this study, the comparison of the D and D* values obtained by the b1 and b2 parameters in addition to the pars petrosa of the temporal bone (including foramen lacerum) of the skull base of the control group and the case group did not show a statistically significant difference. There are a number of reasons for this. First, if the IVIM-DWI uses different b values, it can cause the D, D*, and f values to change. Liao 30 reached the same conclusion in a study of differentiating benign and malignant lymph nodes before and after the treatment of lymphoma. Second, the structure of the skull base is complex, and most of the areas are closely related to bone, muscle, blood vessels, and gas, with the signal thus being easily affected. Third, the comparison of b value selection between the b1 and b2 groups revealed that the b value parameter of the b2 group was too large, the image noise was considerable, the anatomical structure observation was limited, and the selection of region of interest is prone to error. Thus, in this study, the D, D*, and f values of each area of the skull base in the control group and the case group obtained by the b value parameter of the b1 group were more credible than those of the b2 group.

The diagnostic efficacy of D, D*, and f values in each region of the skull base in the control group and the case group obtained by the b value parameter of the b1 group were compared, and the results showed the diagnostic efficacy of the f value in each region of the skull base appeared to be the highest (AUC = 0.908-0.991),the diagnostic efficacy of the D* value was the second highest (AUC = 0.624-0.692). This is consistent with the study of Lai, who found that the AUC of D, D*, and f values of IVIM-DWI in NPC staging was 0.645, 0.828, and 0.905, respectively. Conversely, Zhang indicated an AUC value of D (0.849) to be optimal, which is inconsistent with this study.

Limitations of this study should be considered. First, the sample size of this study was small, and the above results need to be verified with a larger sample size. Second, some measures, such as multiple measurements of ROI, the ROI avoiding large blood vessels and air and so on, were taken in this study to avoid the negative effect of this complexity of the anatomical structure of the skull base. However, the parameter measurement was still affected to a certain extent, and more methods are needed to solve this problem.

In conclusion, the number of b values <200 s/mm2 in IVIM-DWI determines the stability of its application to SBI in NPC. If the b value<200 s/mm2 accounts for more than half of the selected b values, the repeatability of this sequence is extremely high. If the b value is properly selected, the decrease in the D, D*, and f values in the bone and muscle involvement area of the skull base of patients with NPC may become a sign for the diagnosis of SBI. Our findings support the use of IVIM-DWI, a noninvasive method that does not require a contrast agent, in the diagnosis of SBI in NPC.

## Figures and Tables

**Table 1:** The clinical characteristics of recruited SBI in NPC patients

Characteristics	No. of cases (%)
Recruited SBI in NPC patients	36
Male	26(72.22)
Female	10(27.78)
**Age (years)**	
Median	49.5
Range	32-78
**Clinical symptoms and signs**	
Epistaxis	26(72.22)
Hearing impairment	24(66.67)
Nasal obstruction	18(50.00)
Headache	16(44.44)
Facial numbness	5(13.89)
Diplopia and eye symptoms	5(13.89)
Cervical lymph nodes	21(58.33)

SBI, skull-base invasion; NPC, Nasopharyngeal carcinoma.

**Table 2:** Comparison of D, D*, and f values in the b1, b2, and b3 groups of IVIM-DWI

Parameter	b1	b2	b3	b1 vs b2	b1 vs b3	b2 vs b3
				*P*	*P*	*P*
D	1.762±0.628	1.343±0.511	1.809±0.644	< 0.001	0.411	< 0.001
D*	124.075±34.120	110.253±38.197	119.648±35.265	< 0.001	0.194	< 0.001
f	0.293±0.100	0.327±0.128	0.299±0.109	0.002	0.605	0.008

IVIM-DWI, intravoxel incoherent motion diffusion-weighted imaging.

**Table 3:** Comparison of D, D*, and f values in different measurement areas of the skull base with the b1 value in the control group and the case group

Area	Parameter	The control group (n=40)	The case group (n=36)	*P*
Pterygopalatine fossa/processus pterygoidei	D	1.913±0.519	1.808±0.651	0.501
	D*	117.808±31.332	101.084±40.799	0.086
	f	0.391±0.072	0.215±0.075	< 0.001
Sinus sphenoidalis/sellar floor/clivus	D	1.691±0.620	1.442±0.478	0.101
	D*	138.994±27.067	128.227±33.999	0.181
	f	0.306±0.053	0.187±0.078	< 0.001
Pars petrosa of the temporal bone (including foramen lacerum)	D	2.078±0.677	1.628±0.653	0.016
	D*	138.407±31.578	118.801±36.803	0.036
	f	0.356±0.058	0.209±0.051	< 0.001
Corpus of sphenoid bone (including foramen ovale)	D	1.838±0.662	1.371±0.395	0.015
	D*	125.652±25.794	104.831±34.786	0.024
	f	0.354±0.067	0.194±0.072	< 0.001

**Table 4:** Comparison of D, D*, and f values in different measurement areas of the skull base with the b2 value in the control group and the case group

Area	Parameter	The control group (n=40)	The case group (n=36)	*P*
Pterygopalatine fossa/processus pterygoidei	D	1.481±0.484	1.293±0.454	0.145
	D*	108.448±34.534	95.970±47.773	0.257
	f	0.441±0.094	0.238±0.109	< 0.001
Sinus sphenoidalis/sellar floor/clivus	D	1.450±0.576	1.181±0.450	0.058
	D*	123.675±30.233	104.369±40.926	0.041
	f	0.346±0.088	0.195±0.084	< 0.001
Pars petrosa of the temporal bone (including foramen lacerum)	D	1.506±0.608	1.069±0.265	0.002
	D*	117.921±36.659	95.970±36.247	0.030
	f	0.399±0.089	0.214±0.060	< 0.001
Corpus of sphenoid bone (including foramen ovale)	D	1.318±0.425	1.251±0.624	0.661
	D*	119.792±38.589	98.540±34.329	0.073
	f	0.401±0.095	0.236±0.110	< 0.001

**Table 5:** The ROC curve analysis of IVIM parameters in different measurement areas of the skull base with the b1 value

Area	Parameter	AUC	Sensitivity (%)	Specificity (%)	*P*
Pterygopalatine fossa/processus pterygoidei	D	0.587	91.2	30.4	0.269
	D*	0.659	61.8	73.9	0.044
	f	0.949	97.1	87.0	< 0.001
Sinus sphenoidalis/sellar floor/clivus	D	0.574	91.2	32.0	0.344
	D*	0.624	88.2	44.0	0.106
	f	0.908	100	80.0	< 0.001
Pars petrosa of the temporal bone (including foramen lacerum)	D	0.671	44.1	87.0	0.029
	D*	0.671	61.8	73.9	0.030
	f	0.991	100	95.7	< 0.001
Corpus of sphenoid bone (including foramen ovale)	D	0.725	50.0	86.7	0.013
	D*	0.692	94.1	53.3	0.034
	f	0.951	100	86.7	< 0.001

ROC, receiver operating characteristic; IVIM-DWI, intravoxel incoherent motion diffusion-weighted imaging; AUC, area under the curve.
